# Impacts of Continuous and Intermittent Use of Bovine Colostrum on Laying Japanese Quails: Egg Performance and Traits, Blood Biochemical and Antioxidant Status

**DOI:** 10.3390/ani12202811

**Published:** 2022-10-18

**Authors:** Shakir Mokhtarzadeh, Ali Nobakht, Yousef Mehmannavaz, Valiollah Palangi, Hüseyin Eseceli, Maximilian Lackner

**Affiliations:** 1 Department of Animal Science, Maragheh Branch, Islamic Azad University, Maragheh 55, Iran; 2 Department of Animal Science, Agricultural Faculty, Ataturk University, Erzurum 25240, Turkey; 3 Department of Nutrition Sciences, Faculty of Health Sciences, Bandirma Onyedi Eylul University, Bandirma 10200, Turkey; 4 Department of Industrial Engineering, University of Applied Sciences Technikum Wien, Hoechstaedtplatz 6, 1200 Vienna, Austria

**Keywords:** antioxidant, blood parameters, carcass characteristics, egg traits, Japanese quails, bovine colostrum, antibiotics replacement, bovine colostrum (BC)

## Abstract

**Simple Summary:**

Quail is used in cookery, but mainly for its egg production around the globe, and sustainable poultry farming practices have been searched. The use of colostrum (beestings or first milk from cows) in quails’ diet can play an important role in providing probiotics and reducing the need for antibiotics, which, in addition to better quail performance, is effective in reducing environmental impacts. The results of the current research show that the continuous use of bovine colostrum (BC) in laying quails’ diets has beneficial effects on their performance, egg traits, blood indexes and antioxidant status.

**Abstract:**

The Japanese quail (*Coturnix japonica*) is farmed for its eggs and meat across the globe. A series of experiments were conducted to evaluate the effect of the permanent or intermittent use of different levels of BC (bovine colostrum) on the egg performance and traits, carcass characteristics, blood biochemical and antioxidant status of laying Japanese quails. In this study, 200 laying quails were used for a duration of six weeks (week 24 to 30) to measure the selected parameters. Treatments included: (1) control (without BC); (2) 2% continuous BC; (3) 4% BC permanently; and (4) and (5) 2% and 4% BC intermittently (every other week), respectively. According to the results, performance, egg quality, carcass traits, biochemical indices and antioxidant status of BC-fed (continuous and intermittent mode) quails were improved compared to the control-diet-fed birds (*p* < 0.01). Per our observations, quails fed daily with 4% BC had the highest performance, best egg and carcass quality traits, best blood composition and best antioxidant status of serum, although the same parameters were also improved in birds fed intermittently with 4% BC. The final conclusion is that, although quails fed daily with 4% BC showed the best performance, intermittent feeding exerted comparable effects. Therefore, the intermittent-feeding approach could benefit the birds when colostrum preparation is limited due to the high cost of the related process. This approach could improve the economics of poultry breeding while reducing environmental problems, such as antibiotic resistance.

## 1. Introduction

The Japanese quail (*Coturnix japonica*) [1,2] is popular around the world for its high productivity, feed conversion ratio (FCR) and easy breading. Quails have been reared as fighting, singing and decorative birds in Asia from ancient times [3]. They have been used for eggs and meat from the 17th century in Asia and were brought to Europe and North America in the 17th century. In the 20th century, quail farming became industrial. Today, quails are found in all parts of the world [3].

Additional factors that contribute to their popularity are their resistance to poultry diseases, low feed consumption, low space requirement, short gestation, life expectancy and high egg production [4].

In Asia, Japanese quails are typically reared for their egg yield, while in the US and Europe, their meat production is the core driver [5]. In some countries, such as Turkey for instance, Japanese quails are produced for both eggs and meat [5]. Quail meat is important in human diet [6].

Growth performance is affected by feed, temperature [7] and general health status, amongst other factors. Antibiotics in poultry are generally administered to the entire flock for the treatment and prevention of disease, as well as for growth promotion [8]. While antibiotics have been banned as growth promoters in the EU and United States, they are still legally used in several large production countries. Antibiotic usage for disease prevention is permitted in all large poultry-producing countries (give examples) [8]. Antibiotics are applied for the treatment of intestinal infections, such as *colibacillosis, necrotic enteritis* and other diseases generally caused by Salmonella, *E. coli* or *Clostridium* spp. The excessive use of antibiotics by the poultry sector has resulted in the development of antibiotic-resistant strains due to the non-degradable residues and metabolites of these compounds in nature. Many studies have proposed alternatives to antibiotics in recent decades. For instance, El-Hack et al. [9] proposed cinnamon (*Cinnamomum zeylanicum*) oil as a replacement for and as a potential alternative to antibiotics in poultry. Possible replacement agents are probiotics, prebiotics, enzymes, organic acids, herbs, immune stimulants and said essential oils (EO) [9,10,11,12] or red pepper oil [13].

Colostrum, the first mammalian secretion after giving birth, is important in the overall health of newborns domestic animals [14]. Colostrum for mammals may be compared to royal jelly for bees due to its highly nutritive nature. The high energy and protein content, essential fatty acids and multiple vitamins and minerals in colostrum, compared to normal milk, makes it ideal food for newborns [15]. The immunogenic substances and boosters with a non-food nature in colostrum have provided grounds for the improvement of the immune status of infants and their confrontation with pathogens in the period after birth [16]. In the early days after birth, immunoglobulins of the colostrum can easily pass through the intestinal intercellular pores, enter the bloodstream and strengthen a baby’s immune system [17]. A dairy-improved cow can produce about 60 L of colostrum in a period of 3 days after calving, yet its calf cannot consume all the colostrum produced. The unused portion of colostrum is mainly used as normal milk replacement to feed of other calves. Additionally, surplus colostrum could be used in poultry feed. According to a report, the use of 2% fresh cow colostrum in broiler chicken rations (*w/w*) will improve weight gain, reduce losses, and reduce feed costs [18]. In meat-type quails, the use of colostrum powder in up to 5% of quail diets not only improves the performance of quails, but also reduces the oxidation of serum lipids in the blood of quails [19]. Moreover, the use of different levels of colostrum powder in the diets of broilers under normal thermal conditions and heat stress has positive effects on the performance, carcass traits, blood parameters, intestinal morphology and antioxidant status of chickens [20,21]. For a study of hematological parameters in quails depending on their feed, see [22]. Bovine colostrum could be used in fresh, frozen and sour forms, and all have shown advantageous effects on the performance, carcass traits, blood parameters and antioxidant status of chickens [23]. In laying quails, the use of colostrum powder in up to 5% of the diet improved performance, and it was found to enhance egg quality traits and the level of immunity, as well as the antioxidant status of the blood [24]. The purpose of this research was to evaluate the effects of the continuous or intermittent use of bovine colostrum on egg performance and traits, and the blood biochemical and antioxidant status of laying Japanese quails to find out whether breeding economics can be improved by such an intermittent-feeding mode. For a study of feed withdrawal strategies in general, see [25].

## 2. Materials and Methods

Ethical Protocol (no. 1365-IAU) was approved by the experimental animal ethics committee of the Islamic Azad University, Maragheh branch, Iran.

### 2.1. Animals and Housing Conditions

We used 200 laying quails (from week 24 to 30) in the current study. This study had a completely randomized design and was performed in 5 treatments with 4 replicates (10 quails per replicate). The treatments included 5 groups:

(1) control (without bovine colostrum (BC)),
(2) 2% of BC permanent administration,
(3) 4% of BC permanent administration,
(4) 2% of BC intermittent administration (every other week)
(5) 4% of BC intermittent administration (every other week)

Quails were randomly assigned to treatments and replications of experimental units. Diets were formulated according to the NRC (1994) quail nutrition guide, with a base of corn and soybean meals using UFFDA’s diet-formulation software (Table 1). Colostrum required for the experiments was produced in the first 6 h, and a third calving of a Holstein cow was prepared in a sufficient quantity and stored at −20 °C until the start of the experiment. Bovine colostrum (BC) [15] is high in nutritional value. Colostrum is the initial milk secreted by mammals following parturition [15]. Used colostrum contained 22% CP, 14.8% fat, 2.81% lactose and 42 (g/L) immunoglobulin G, which was added in the form of a spray to the experimental feeds and mixed for homogenization.

At the time of use, BC was completely thawed and mixed thoroughly with the whole diet. During the experiment, water and feed were provided ad libitum. In experimental groups 4 and 5, quails were fed a diet containing cow colostrum for one week and a control diet for another week. The temperature and humidity of the experimental room were controlled, and the lighting program used during the experimental period included 8 h of darkness and 16 h of light.

### 2.2. Performance Data Collection

Quail eggs were collected on a daily basis, while feed intake was measured weekly, and mortality was recorded and applied on the base of hen day. Egg mass was determined by multiplying the percentage of the daily egg-production percentage by the average weight of the eggs. The feed conversion ratio was calculated by dividing egg mass production by feed consumption. At the end of the experimental period, 4 eggs were collected from each experimental unit to measure the egg traits (egg shape index, egg albumin percentage, egg yolk index, egg-shell percentage, yolk color and Haugh unit), where the Haugh unit describes the egg protein quality measured by the height of its albumen (egg white).

### 2.3. Carcass Traits

In order to determine the carcass traits, two quails were slaughtered from each of the experimental units and the carcass percentage, as well as the percentage of intestines, liver, spleen, gizzard, breast and thighs was determined (by weighting) [27].

### 2.4. Blood Biochemical and Antioxidant Status

To determine the blood and antioxidant parameters, blood samples were taken from quails (two quails in each replicate) at the end of the experiment, and the parameters were measured according to the relevant protocols. Approximately 5 mL of blood samples were collected from randomly chosen birds through the brachial vein. The blood samples were subjected to centrifugation to obtain serum and to measure the blood lipids (cholesterol, triglyceride, low- and high-density lipoprotein). Kit packages (Pars Azmoon Company; Tehran, Iran) were used to determine the blood biochemical parameters using an Anision-300 auto-analyzer system [28].

### 2.5. Statistical Analysis

This study was based on a completely randomized design. Data normality was tested using SAS [29]. The data (production performance, intestinal morphology and composition of fecal nutrients) was subjected to the PROC GLM procedure of SAS software. Data were log-transformed before analyzing to compensate for any unequal variances. Duncan’s test, at a 5% level of probability, was used to compare the means.

## 3. Results

### 3.1. Performance

The effects of using different levels of colostrum administered continuously and intermittently on the performance of laying quails are shown in Table 2. Different methods of using bovine colostrum compared to the control diet improved the laying performance of quails (*p* < 0.01). The highest values were related to egg weight (12.48 g) and egg mass production (8.55 g) with the continuous use of 4% colostrum, while the highest percentage of egg production (68.35%) was obtained using 4% colostrum on a weekly basis. All experimental treatments improved the feed conversion ratio compared to the control. There was no significant difference in the amount of daily feed intake between the experimental groups compared to the control (*p* > 0.05).

The effects of different concentrations of BC and its application technique on the quality characteristics of Japanese-laying-quail eggs are shown in Table 3. The levels of and manner of using BC significantly affected all quality traits of quail eggs compared to the control group (*p* < 0.01). Among the experimental treatments, the continuous use of 4% colostrum, compared to the other levels and treatment methods, had more positive effects on the quality traits of quail eggs. Thus, birds fed continuously with 4% BC showed the highest egg white, yolk and shell percentages, as well as the highest indices related to egg-shell thickness, yolk color and Haugh unit. However, the intermittent use of bovine colostrum in diets compared to the control group had positive effects on the quality traits of the studied eggs. The highest percentage of egg yolk was observed in the control group, which was due to a large decrease in the percentage of egg white and shell in the control group compared to the experimental groups.

### 3.2. Carcass Traits

The effects of different concentrations of bovine colostrum and its application technique on the carcass traits of Japanese laying quails are reflected in Table 4. The use of bovine colostrum had positive effects on quail carcass traits (*p* < 0.01). The highest percentages of carcasses, intestines, gizzard, breast and thighs were observed in quails continuously fed with 4% bovine colostrum, although the intermittent use of bovine colostrum, in comparison with the control, improved the carcass traits of quails in most cases. Quails were found to exhibit the highest percentage of liver and kidneys (by mass) in the control group. The other species were broilers (see [12]) that were examined on laying hens.

### 3.3. Blood Biochemical and Antioxidant Status

The effects of varying concentrations of BC and its application technique on quail blood indices are shown in Table 5. Both the concentration and the method of application significantly affected the blood parameters of quails (*p* < 0.01). Serum antioxidant indices (superoxide dismutase and malondialdehyde) were improved by using BC compared to the control diet. Treatments had stimulatory effects on blood superoxide dismutase, while the effects were inhibitory on malondialdehyde levels. Blood protein indices (albumin and total protein) increased by using BC in quail diets. BC had additive effects on lipid-related compounds (LDL and triglycerides). Nonetheless, blood mineral levels (calcium, phosphorus [as phosphate] and magnesium) were lower in all groups of 7 BC-fed birds.

## 4. Discussion

Colostrum, rich in nutrient and non-nutrient compounds, can significantly improve the production performance of quails without a substantial increase feed intake in the birds. On the one hand, bovine colostrum, in comparison to normal milk, contains multiple folds of essential amino acids, fats and important fatty acids and fat-soluble vitamins, as well as minerals [15]. On the other hand, bovine colostrum is rich in immunogenic substances and vital antibodies [16]. Important nutrients of the BC would ensure the optimal utilization of feed, increasing the performance of quails and optimizing production requirements, while immune-boosting molecules improve the overall health of birds. Thus, BC-fed quails demonstrated the best performance compared to the control group. Higher percentages of intestines in BC-fed birds show increased numbers of absorption cells in the intestine, improving the efficiency of nutrient uptake compared to the control group. The continued improvement in the performance of the one-week-receiving groups among bovine colostrum, compared with the control group, suggests that some of the nutrients in the colostrum may be stored in tissues, exerting long-term benefits. The improved performance in intermittent-BC-fed quails could be explained by the general immune-boosting and optimal feed-utilization attributes of the colostrum. Our results agree with those reporting the beneficial effects of different BC levels on the overall health and performance of several poultry species [18,19,20,21,22,23] (e.g., in broilers) [12]. No change in the amount of daily feed consumption of quails in experimental diets is contrary to the report of Bayril et al. [24]. Bayril et al. [24] reported that the use of up to 5% of bovine colostrum powder in the diet of laying quails improved selected production traits, while increasing feed intake. The reason for this observed difference could be due to the form and amount of colostrum used, the production stage of quails and other components of the diets. The increase in egg albumin percentage is possibly due to the ideal protein and amino acids content of colostrum. Improvements in yolk index could be due to the content of fat and essential fatty acids in colostrum. Yolk color difference is possibly related to the presence of vitamin A and carotene in colostrum. The improved percentage (weight) and thickness of eggshell is associated with high levels of minerals, especially calcium, present in bovine colostrum. Most of the nutrients in colostrum are estimated to be several times higher than normal milk [30]. The decrease in yolk percentage was due to the increase in albumin and shell percentages. Our results about the effect of BC on the quality traits of quail eggs are in agreement with the observations of Bayail et al. [24] about the improved thickness and weight of quail eggs in colostrum-fed birds. The relative improvement of egg quality traits with the intermittent use of bovine colostrum in quail diets, compared to the control group, indicates that some of the nutrients in colostrum have a long-term storage capacity in the body and support the production of high-quality eggs for a long time. The richness of bovine colostrum in various nutrients, especially proteins and amino acids, has increased the percentages of carcasses, intestines, gizzards, breast and thighs in quails. The relatively smaller liver mass in our study may be indicative of the presence of sufficient immunogenic substances of the BC which, upon consumption, benefits the birds. Our observations on the beneficial effects of BC on carcass traits agreed with those of Gorbannejad-Parapary et al. [20,21]. Increasing the percentage of intestines by using bovine colostrum in the diet, compared to the control group, can increase the number of absorption cells in the intestine and improve the efficiency of nutrient uptake; thus, it can also improve the carcass traits. Bovine colostrum, which is rich in antioxidants, such as vitamins A and E, increased the concentration of superoxide dismutase (an antioxidant enzyme) in the birds’ blood. Moreover, hepatic malondialdehyde showed that characteristic effects and oxidative activity was significantly lower in BC-fed birds due to its antioxidant properties. The present findings are consistent with the report of Gorbannejad-Parapary et al. [21]. Presumably, the high protein content of BC led to higher albumin and total protein in the blood of quails. Gorbannejad-Parapary et al. [20] reported rather comparable total protein results with an insignificant effect on albumin in the blood of chickens. The results of LDL and TG concentrations of BC-fed quails, compared to the control birds, did not agree with the observations of Gorbannejad-Parapary et al. [21], in which they reported lower LDL in the blood of chickens. The source of these variations could be type of bird, the source of colostrum and the difference in the type of production and composition of diets. Lower blood calcium, phosphorus and magnesium concentrations of B-fed laying quails may be due to an increased production of birds accumulating large amounts of these nutrients in their products. This may also be due to the higher metabolism of birds with an increased production. Elbaz et al. [25] studied feed withdrawal (FW) in Japanese quails and found that FW, either for 1/2 or a 1/3 of the day, can be a promising dietary strategy for improving meat quality. Combining FW with BC could be an interesting route to explore in future research.

## 5. Conclusions

Based on our results, the application of BC up to 4% in the stage of laying quails’ diet can improve performance, egg quality traits, carcass traits, immunity, and antioxidant status, as well as blood indicators. The higher efficiency of quails with an intermittent use of bovine colostrum makes the approach cost effective, as well as feasible, when the supply is limited, although continuous use is recommended. Due to the novelty of using colostrum in poultry diets, more basic and applied research is recommended.

## Figures and Tables

**Table 1:** The ingredients and chemical composition of the diets used in the experiments.

Feeds Ingredients (%)		Colostrum %	
	0	2	4
Corn	50.00	49.00	48.00
Wheat	23.73	22.18	20.63
Soybean meal—44%	16.31	14.75	13.21
Soybean oil	0.24	0.36	0.48
Colostrum	0	2.00	4.00
Oyster shell	7.83	7.83	7.81
Dicalcium phosphate	1.11	1.10	1.10
Salt (NaCl)	0.28	0.28	0.27
Vitamin premix ^1^	0.25	0.25	0.25
Mineral premix ^2^	0.25	0.25	0.25
Calculated composition			
Metabolizable energy (Kcal/kg)	2800	2800	2800
Crude protein (%)	14.00	14.00	14.00
Ca (%)	3.28	3.28	3.28
Available phosphor (%)	0.31	0.31	0.31
Crude fiber (%)	2.86	3.28	3.98
Sodium (%)	0.15	0.15	0.15
Lysine (%)	0.63	0.63	0.63
Methionine + Cysteine (%)	0.55	0.55	0.55
Tryptophan (%) [26]	0.18	0.18	0.18

^1^ Description of the vitamin premix per kg of diet: vitamin A (retinol), 8,500,000 IU (international units); vitamin D3 (Cholecalciferol), 2,500,000 IU; vitamin E (tocopheryl acetate), 11,000 IU; vitamin k_3_, 2200 mg; thiamine, 1477 mg; riboflavin, 4000 mg; pantothenic acid, 7840 mg; pyridoxine, 7840 mg; cyanocobalamin, 10 mg; folic acid, 110 mg; choline chloride, 400,000 mg. ^2^ Mineral premix per kg of diet: Fe (FeSO_4_.7H_2_O, 20.09% Fe), 75,000 mg; Mn (MnSO_4_.H_2_O, 32.49% Mn), 74.4 mg; Zn (ZnO, 80.35% Zn), 64.675 mg; Cu (CuSO_4_.5H_2_O), 6000 mg; I (KI, 58% I), 867 mg; Se (NaSeO_3_, 45.56% Se), 200 mg.

**Table 2:** Effects of using bovine colostrum on the performance of laying quails.

Performance	Egg Weight (g)	Egg Production (%)	Egg Mass (g/(h*d))	Feed Intake (g/(h*d))	Feed Conversion Ratio
Treatments					
0	11.53 ^c^	49.23 ^c^	5.69 ^d^	25.22	4.41 ^a^
2C	12.34 ^ab^	67.40 ^ab^	7.57 ^bc^	25.99	3.33 ^b^
4C	12.48 ^a^	68.35 ^ab^	8.55 ^a^	25.91	3.30 ^b^
2I	11.86 ^bc^	61.29 ^b^	7.05 ^c^	25.56	3.39 ^b^
4I	11.97 ^abc^	70.51 ^a^	7.71 ^b^	26.08	3.23 ^b^
SEM	0.19	2.63	0.19	0.32	0.13
*p*-value	0.0197	0.0003	0.0001	0.1084	0.0001

Values in the same column which do not share a common superscript differ significantly (*p* < 0.01). 0 = Control, 2C = 2% Bovine colostrum continuously. 4C = 4% bovine colostrum continuously, 2I = 2% bovine colostrum a week in between, 4I = 4% bovine colostrum a week in between. SEM = standard error of the mean. g/(h*d) = grams per hen and day.

**Table 3:** Effects of using bovine colostrum on some egg traits of laying quails.

Egg Traits	Egg Albumin (%, Mass)	Egg Yolk Index	Egg Yolk (%, Mass)	Egg Shell (%, Mass)	Shell Thickness (mm)	Yolk Color	Haugh Unit
Treatments							
0	60.04 ^b^	60.82 ^ab^	30.75 ^a^	8.52 ^c^	0.238 ^c^	3.48 ^c^	95.65 ^b^
2C	58.26 ^a^	61.90 ^b^	28.09 ^b^	9.31 ^ab^	0.255 ^bc^	4.90 ^b^	96.76 ^ab^
4C	65.69 ^b^	65.85 ^a^	28.86 ^b^	9.43 ^a^	0.290 ^a^	7.20 ^a^	98.34 ^a^
2I	61.90 ^b^	60.66 ^ab^	28.85 ^b^	8.81 ^bc^	0.248 ^bc^	4.53 ^b^	96.09 ^b^
4I	64.45 ^b^	62.24 ^ab^	27.26 ^b^	8.94a ^bc^	0.278 ^abc^	5.37 ^b^	96.87 ^ab^
SEM	1.96	1.14	0.54	0.17	0.01	0.30	0.71
*p*-value	0.1279	0.0354	0.0051	0.0084	0.0084	0.0001	0.0330

Values in any of the columns without a common superscript differ significantly (*p* < 0.01). 0 = Control, 2C = 2% bovine colostrum continuously. 4C = 4% bovine colostrum continuously, 2I = 2% bovine colostrum a week in between, 4I = 4% bovine colostrum a week in between.

**Table 4:** Effects of using bovine colostrum on carcass traits of laying quails. (%).

Carcass Traits	Carcass	Intestine	Liver	Spleen	Gizzard	Breast	Thighs
Treatments							
0	64.12 ^c^	7.88 ^b^	6.66 ^a^	0.31	3.71 ^b^	30.92 ^bc^	16.86 ^c^
2C	70.02 ^ab^	8.01 ^b^	4.07 ^b^	0.17	4.22 ^b^	33.06 ^ab^	19.24 ^ab^
4C	73.63 ^a^	11.46 ^a^	3.69 ^b^	0.16	5.14 ^a^	36.19 ^a^	20.75 ^a^
2I	68.22 ^bc^	8.26 ^b^	3.77 ^b^	0.18	3.96 ^b^	28.47 ^c^	18.55 ^bc^
4I	70.31 ^ab^	9.66 ^b^	3.92 ^b^	0.16	4.03 ^b^	31.09 ^c^	18.90 ^b^
SEM	1.55	0.56	0.23	0.44	0.30	1.33	0.59
*p*-value	0.0092	0.0018	0.0001	0.4442	0.0356	0.0117	0.0059

Values in the same column that do not share a common superscript differ significantly (*p* < 0.01). 0 = Control, 2C = 2% bovine colostrum continuously. 4C = 4% bovine colostrum continuously, 2I = 2% bovine colostrum a week in between, 4I = 4% bovine colostrum a week in between.

**Table 5:** Effects of using bovine colostrum on blood parameters of laying quails.

Blood Indexes	SOD (g/dl)	Uric Acid (g/dl)	Albumin (g/dl)	Protein (g/dl)	ALT (mg/dl)	AST (mg/dl)	MDA (g/dl)	LDL (mg/dl)	TG (mg/dl)	Ca (mg/dl)	P (mg/dl)	Mg (mg/dl)
Treatments %												
0	1.37 ^d^	4.34	1.30 ^b^	3.35 ^c^	32.50	239.75	4.13 ^a^	38.30 ^c^	790.50 ^c^	28.60 ^a^	7.35 ^a^	3.45 ^a^
2C	2.71 ^b^	4.33	1.58 ^a^	4.05 ^b^	33.50	251.75	2.73 ^b^	50.55 ^a^	983.50 ^ab^	25.45 ^ab^	5.75 ^b^	2.83 ^b^
4C	3.50 ^a^	4.56	1.63 ^a^	4.58 ^a^	34.35	265.50	1.90 ^c^	49.55 ^ab^	1010.75 ^a^	23.13 ^b^	5.83 ^b^	2.69 ^b^
2I	1.88 ^c^	4.42	1.33 ^bc^	3.63 ^bc^	32.25	242.25	1.55 ^c^	42.95 ^bc^	884.00 ^bc^	22.60 ^b^	5.36 ^b^	2.56 ^b^
4I	2.36 ^b^	4.04	1.43 ^b^	3.95 ^b^	32.25	237.25	1.83 ^c^	41.53 ^c^	980.00 ^ab^	23.85 ^b^	5.59 ^b^	2.68 ^b^
SEM	0.14	0.32	0.07	0.17	2.61	1.79	0.23	2.24	35.98	1.35	0.32	0.10
*p*-value	0.0001	0.8409	0.0085	0.0020	0.9746	0.4270	0.0001	0.0060	0.0031	0.0423	0.0040	0.0001

Values in any of the columns without common superscript differ significantly (*p* < 0.01). 0 = Control, 2C = 2% BC continuously. 4C = 4% BC continuously, 2I = 2% BC a week in between, 4I = 4% BC a week in between. SOD = superoxide dismutase, ALT = alanine aminotransferase, AST = aspartate aminotransferase, MDA = malondialdehyde, LDL = low-density lipoprotein.

## Data Availability

The data presented in this study are available in this article.
